# Summary report of the 1st MOVE symposium in Málaga from 24-27th October 2023 - Foster the European mobility for young scientists in extracellular vesicles research

**DOI:** 10.20517/evcna.2024.09

**Published:** 2024-02-26

**Authors:** Mia S. C. Yu, Tanja V. Edelbacher, Christian Grätz, Dapi M. Chiang, Marlene Reithmair, Michael W. Pfaffl

**Affiliations:** ^1^Chair of Animal Physiology and Immunology, School of Life Sciences, Technical University of Munich, Freising 85354, Germany.; ^2^Division of Functional Microbiology, Institute for Microbiology, Center for Pathobiology, Department for Biological Sciences and Pathobiology, University of Veterinary Medicine Vienna, Vienna 1210, Austria.; ^3^Institute of Human Genetics, University Hospital, LMU Munich, Munich 80336, Germany.; ^4^Department of Biomedicine, University of Basel, Basel 4031, Switzerland.; ^#^Authors contributed equally.

## INTRODUCTION

**Figure 1:**
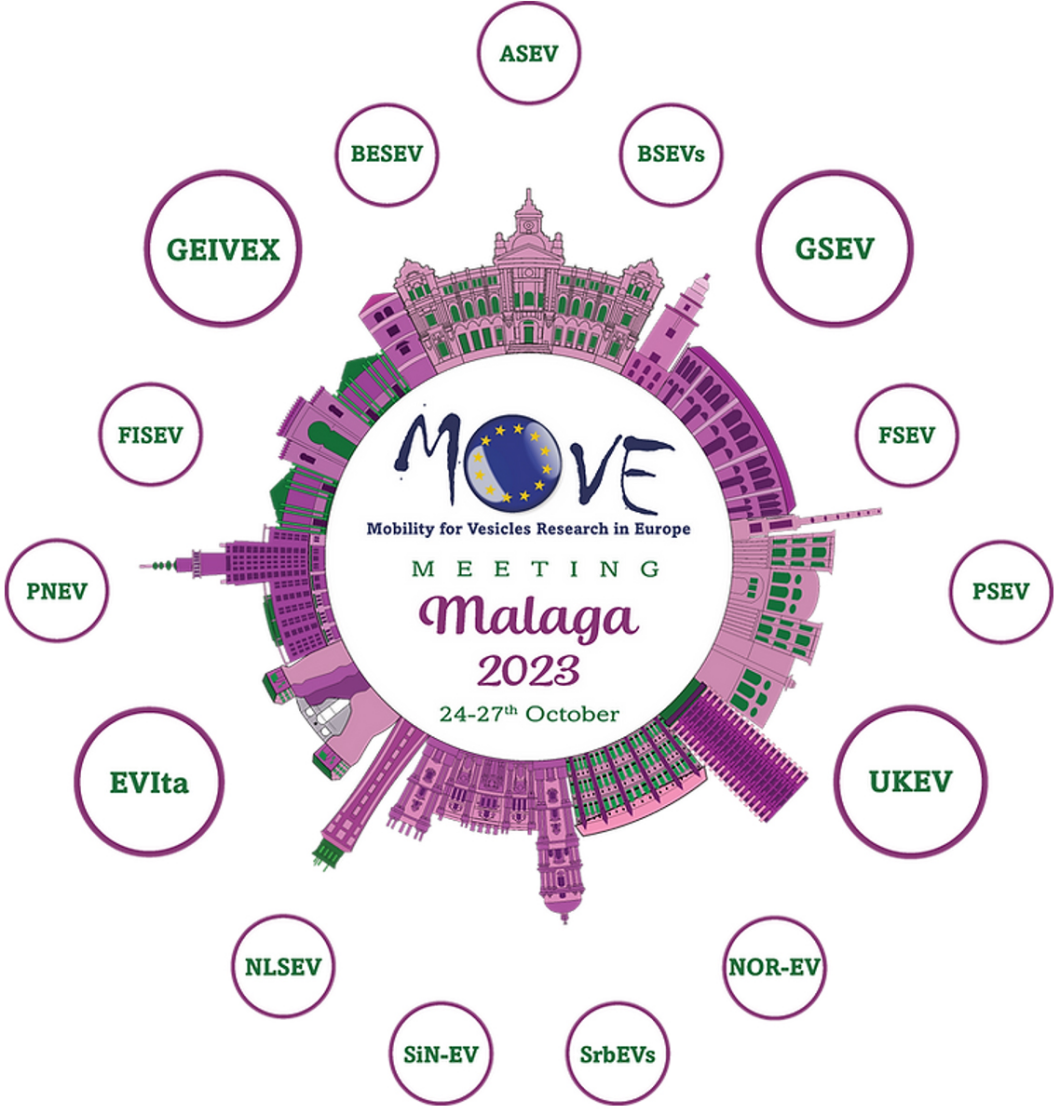
The MOVE 2023 logo was designed by Maja Kosanović, president of the Serbian EV Society (SrbEVs). The colors were inspired by the prominent colors green and purple of the Málaga city coat of arms, while the center depicts famous buildings that can be found in Málaga. The bigger circles display the societies that were involved in the organization of the meeting; the smaller circles represent the other European societies that joined the MOVE meeting. The illustration of the city can also be interpreted as a cell shedding EVs, represented by the different European EV societies.

The 1st “Mobility for Vesicle Research in Europe” - MOVE - symposium took place in the picturesque Málaga, the capital of the Costa del Sol and the Mediterranean sun city of Spain. The principal organizer, GEIVEX, supported by the societies GSEV, UKEV, and EVIta, spared no effort to organize a fabulous international symposium. More than 300 participants, mainly members from 16 joining European Extracellular Vesicle (EV) societies (ASEV, BESEV, BSEVs, CzeSEV, EVIta, FISEV, FSEV, GEIVEX, GSEV, HSEV, NLSEV, NOR-EV, PNEV, PSEV, SrEVs, and UKEV; see Figure 1), attended this interdisciplinary conference held at the Faculty of Law of the University of Málaga. The diversified and multidisciplinary program of the four days covered five invited keynote talks given by Hernando de Portillo, Antonella Bongiovanni, Martine J. Smit, Fedor Berditchevski, and Benjamin Winkeljann, and 76 oral presentations and 157 posters by young European EV scientists. The sessions were framed by an industrial exhibition that gave the scientists the opportunity to have fruitful discussions with industrial partners and find out about new devices and products in EV research on the market. The MOVE Fellowship Program enabled several young scientists to participate in an exchange program at collaborating facilities, resulting in network promotion and projects enriching the EV field. Eight of them were able to present their experiences gained with the aid of the MOVE Fellowship program, closing the symposium. The symposium dinner located at Baños del Carmen was definitely one of the conference’s highlights. Surrounded by the beach and the sea, the participants enjoyed networking while savoring delicious Spanish tapas and, afterward, rocking the dance floor. The 1st MOVE symposium was absolutely a great success, and Málaga, as a lively, young, and creative city with its numerous historical attractions and nightlife, proved to be the perfect event location.

## THE PRESENTATIONS

The presentations of the young EV scientists were introduced and framed by five internationally renowned keynote speakers.

### Keynote speaker I: Hernando del Portillo

After a welcome by Prof. Isabel Lucena, Prof. Juan Jose Hinojosa Torralvo, and Prof. Zaida Díaz Cabiale, the meeting opened with an engaging lecture by Hernando del Portillo with the title “*EVs and Parasitic Diseases: Let’s MOVE-on”* (see publication in this issue). After pointing out the impact of parasites on global health, he provided examples of the importance of EVs in parasites. For instance, the drug resistance of *Leishmania*, a parasite that is transmitted by sandflies and has infected millions of patients globally, can be transmitted via EVs^[1]^. *Heligmosomoides polygyrus*, an intestinal nematode of rodents, can utilize EVs to transmit microRNAs (miRNAs) to its host cells, which results in the downregulation of genes involved in immunity and inflammation^[2]^. EVs released by nanotubes from *Trypanosoma brucei*, a kinetoplastid that causes sleeping sickness, alter the membrane of red blood cells, resulting in anemia^[3]^. Portillo’s talk then focused on his research on the globally prevalent *Plasmodium vivax*, one of the parasites that causes malaria. *P. vivax* is transferred by mosquitos in the form of a sporozoite. In the new host, the parasite multiplies in hepatocytes. After lysis of the infected hepatocytes, *P. vivax* enters red blood cells, where it develops into a sexual ring stage and eventually destroys the red blood cells or is taken up by a mosquito. In the liver, *P. vivax* can persist for months to years, and 90% of all infections are asymptomatic. Portillo’s lab took advantage of the fact that in chronic infections, 98% of the parasites are found in the spleen, since infected red blood cells cannot pass through it. To obtain infected cells from untreated patients with chronic disease, the group isolated red blood cells from spleens resected from accident victims and checked for the presence of *P. vivax*. Dosing of human spleen fibroblasts with EVs originating from those infected red blood cells resulted in the upregulation of ICAM1, an endothelial receptor to which plasmodium variant interspersed repeats (VIR) proteins expressed in infected red blood cells can attach^[4,5]^. This was confirmed in a functional assay using Bay 11-7082, a broad-spectrum inhibitor that reduces the expression of ICAM-1 and NF-κB, among others^[6]^. These findings further indicate that the spleen is one of two suspected niches for cryptic infections with *P. vivax* outside the liver, the second niche being the bone marrow^[7]^. *P. vivax* presence was also confirmed in the bone marrow of infected patients, leading to dyserythropoiesis^[8]^. Finally, Portillo showed exciting unpublished results from organ-on-a-chip models concerning the bone marrow cryptic niche^[9]^.

### Keynote speaker II: Antonella Bongiovanni

At the beginning of her lecture titled “*Harnessing Nature’s nanoSecrets: Microalgal-Derived Extracellular Vesicles as Bio-Based Nanoparticles for next-level Pharmaceutical and Cosmetic applications”*, Antonella Bongiovanni gave a short introduction to the history of the EV field. Here, she highlighted that Darwin had already suggested that all cells were capable of shedding small particles that migrate through the body. Today, the EV field is gaining more and more importance, and important research connected to it has even been awarded the Nobel Prize in Medicine in 2013. EVs exist throughout all domains and enable interspecies communication and interaction. Like other EVs, plant EVs measure 30-150 nm in diameter and are equipped with molecular markers in the form of membrane trafficking proteins and proteins derived from the trans-golgi network (TGN) and the multivesicular body (MVB), such as TET8 and TET9^[10]^. Moreover, the secretion via MVB is enhanced in response to a bacterial or parasite infection. EV cargo can include cell wall-building enzymes that may be involved in plant growth and development, as well as pathogen defense proteins that can be delivered to the site of infection^[11]^. In the marine ecosystem, EVs originating from the marine phototrophic bacteria *Prochlorococcus* make up 10^5^-10^6^ vesicles/ml of seawater^[12]^. Blooms of the cosmopolitan algae *Emiliana huxleyi* are limited by EVs which are produced upon induction by viruses that use them to sustain efficient infectivity and propagation^[13]^. Recently, EVs have gained importance as natural drug delivery systems - either through native or engineered EVs, which makes them highly interesting for companies. These EVs mainly stem from mammalian cells whose production is neither scalable nor economically sustainable^[14,15]^. The alternative presented by Bongiovanni are EVs obtained from microalgae such as the marine *Tetraselmis chuii* that can grow in large-scale photobioreactors and are independent of seasonal fluctuations while many of them are even edible^[16]^. Microalgae EVs were isolated using tangential flow filtration, and the algosome identity was characterized by dynamic light scattering, NTA, Western Blot, cryo-EM, scanning electron microscopy, atomic force microscopy, and on lipidomic and proteomic level^[16,17]^. Nanoalgosomes were furthermore found to be very stable at high and low pH, high temperatures (up to 60 °C) and osmotic shock while being biocompatible^[18]^. Additionally, it could be shown in *Caenorhabditis elegans* that microalgal EVs can be taken up *in vivo*^[19]^. Bongiovanni also showed results of mice experiments that revealed no toxicity and gave some insight into the biodistribution. The uptake is an active process, although the exact mechanism underlying it is still unknown. The cultivation in a renewable and scalable bioprocess paired with a highly versatile loading capacity makes microalgal EVs interesting for pharmaceutical and cosmetic applications as the production is GMP compliant, meaning that it is easily expandable and adaptable to demand^[16,17]^.

### Keynote speaker III: Martine J Smit

The third keynote lecture was given by Martine J. Smit with the title *“Convergence of G protein-coupled receptor (GPCR) and extracellular vesicle biology”*. Cellular transmembrane receptors, particularly G protein-coupled receptors (GPCRs), function as antennae on the plasma membrane, gathering external information. EVs have expanded intercellular communication by transporting functional receptors, ligands, and signaling proteins. Recent findings suggest the involvement of both GPCRs and EVs, presenting opportunities for future therapeutic advancements at their intersection^[20]^. GPCRs, notably the chemokine receptor CXCR2 and muscarinic M1 receptor, exert influence on multivesicular body (MVB) maturation by affecting intraluminal vesicle (ILV) budding^[21-23]^. Studies suggest that GPCR signaling pathways, such as the autocrine activation of MVB-resident sphingosine-1-phosphate (S1P) receptors, aid in cargo sorting into ILVs through Gβγ subunit-dependent activation of Rho-family GTPases and actin remodeling at the limiting membrane^[24]^. Additionally, the muscarinic M1 receptor triggers ILV formation by inducing diacylglycerol production at the MVB membrane, subsequently activating protein kinase D^[21,22]^. GPCR signaling pathways impact various cellular processes, influencing MVB fusion with lysosomes or the plasma membrane. Specifically, GPCRs trigger calcium-dependent exosome secretion through pathways involving phospholipase C, illustrating cell type-specific mechanisms in exosome release^[20]^. Some GPCRs not only regulate EV production but also influence their functions. For instance, GPCRs like metabotropic glutamate receptor 1 (GRM1) in melanoma cells^[25]^ and CXCR1 in hepatocytes alter the cargo of their released EVs^[23]^, impacting their pro-migratory or proliferative effects. The precise mechanism by which GPCRs influence EV cargo - whether directly sorting cargo or reflecting broader changes in cellular content - remains an area requiring further exploration. Abnormal G-Protein Coupled Receptor (GPCR) activity is linked to various diseases, including cancer, where GPCR signaling plays a role in cell communication via exosomes^[26]^. These nanovesicles, released more abundantly by tumor cells, and potentially influenced by altered GPCR signaling, aid in preparing distant sites for metastasis by affecting recipient cells^[26]^. Human cytomegalovirus (HCMV) uses viral GPCRs like US28 and UL33, impacting cancer, particularly in glioblastoma^[27,28]^. Both receptors influence cancer pathways; however, US28, found in MVBs, sorts into exosomes, aiding HCMV in evading immune responses by hindering specific pathways, which is crucial in virus-host interactions^[27,28]^. Camelid heavy-chain-only antibody fragments, known as nanobodies, have emerged as potent tools in GPCR research. They assist in understanding GPCR signaling and intracellular processes, such as signaling shut-off and internalization, offering the potential for targeted therapies with reduced side effects^[29,30]^. Additionally, nanobody-conjugated photosensitizers selectively eliminated US28-expressing glioblastoma cells using targeted photodynamic therapy, presenting a novel approach that exploits GPCR targeting for precise cancer therapy^[31]^. Inspired by nanobody research, Martine J Smit’s group initiated an investigation into EVs and discovered the localization of the HCMV-encoded receptor, US28, within MVBs, particularly colocalizing with CD63. This localization suggests its potential role in HCMV's immune evasion and chemokine scavenging^[27,32]^. In GPCR signaling, EVs emerge as crucial carriers for proteins and hydrophobic ligands, impacting long-range transport and cellular signaling processes. These EVs facilitate breast cancer cell metastasis by sorting and transferring Wnt11-loaded vesicles derived from fibroblasts, thus stimulating cancer cell migration and metastasis^[33]^. Furthermore, EVs contain various hydrophobic molecules such as S1P, prostaglandins, and endocannabinoids, significantly influencing cancer progression and metastasis through signaling mechanisms^[20,34-37]^. Acting as independent biofactories, EVs contain enzymes and precursors that generate GPCR ligands like cAMP, S1P, activated factor X (FXa), and protease-shed ligands, indicating their tissue-specific GPCR activation potential^[20]^. The heightened activation of FGF2 and IL-6 by TGF-β confined within EVs in cancer-triggered fibroblasts and mesenchymal stem cells residing in tumors suggests differences compared to free-form TGF-β^[38]^. EV-carried GPCRs may serve as potential biomarkers and therapeutic tools.

### Keynote speaker IV: Fedor Berditchevski

In the following keynote lecture titled “*Tetraspanins and extracellular vesicles: together and forever”*, Fedor Berditchevski highlighted significant findings from research focused on tetraspanins and EVs. Tetraspanins, a class of transmembrane proteins, serve as abundant markers on EVs, particularly eukaryotic EVs^[39]^. The presented insights emphasize the critical role of EVs in regulating cell-to-cell communication within the immune microenvironment of breast cancer. It has already been known that EVs can act as a messenger among cancer cells^[40]^ and that they can mediate cell migration^[41]^. The presented study specifically investigated the potential impact of EVs on immune cell recruitment, influencing the tumor microenvironment (TME). Through meticulous PBMC migration assays, transwell assays, and flow cytometry, the research revealed a distinct response solely from CD19^+^ cells to media conditioned by breast cancer cells. Further insights were gained through the application of *Bordetella pertussis* toxin PTX, known for its capability to impede the migration of B-cells - an activity reliant on classical chemokines. The receptors responsible for chemokine sensing were also found to respond to PTX. PTX is recognized for its role in modulating G-protein activity^[42]^, thereby inducing elevated cAMP levels and influencing various cellular processes. Notably, lipid signaling molecules such as S1P, oxysterols, and eicosanoids were highlighted as ligands for their involvement in diverse cellular functions, including immune regulation and inflammation^[43,44]^. Out of those, Berditchevski focused on the binding of oxysterols to liver X receptor (LXR) and the hypothesis that LXR-dependent transcriptional network is active in migrating B cells was proposed. RNA-seq confirmed an LXR signature (NR1H3) among the top three differentially regulated genes, and it was shown that NR1H3 transcriptional activity correlates with B-cell migration. As TSPAN6 regulates the oxysterol-composition of EVs which then affect the LXR pathway, EVs can regulate B cell migration^[45]^. Similarly, the expression of the tetraspanin protein CD151 by tumor cells has a notable impact on shaping the immune microenvironment by modulating the recruitment of monocytes to cancerous tissues. Consequently, targeting this CD151-mediated pathway emerges as a novel and deliberate approach to alter the immune landscape in inflammatory breast cancer. This again highlights the importance of tetraspanins and their potential as therapeutical targets^[46]^.

### Keynote speaker V: Benjamin Winkeljann

In the symposium closing lecture titled “*Escaping the Endosome: Overcoming the Final Barrier for Effective Drug Delivery”*, Benjamin Winkeljann focused on the difficulties faced for effective drug delivery and approaches to conquer them successfully (see publication in this issue). Physiological barriers for drug delivery can be local (e.g., lung, tumor site, eye, *etc.*) or systemic (e.g., gastrointestinal, intravenous) and affect each step on the way to successful drug delivery from stomach over immune cells, blood filtration, cellular membrane, endosomal compartment, and cell nucleus to the final target^[47]^. Moreover, there are cell-specific differences such as membrane porosity, trafficking routes, and kinetics/time or endosomal size variations. These differences call for different drug carriers. Lipid nanoparticles such as the COVID-19 vaccine deliver drugs via membrane fusion through charge interactions, while less than 2%-3% manage to escape the endosome^[48]^. Poly-or Micelleplexes, on the other hand, can deliver drugs either via the proton sponge effect or membrane destabilization, while less than 1%-2% manage to escape the endosome^[47]^. The back fusion of EVs with the endosomal membrane and an escape rate of approximately 25% depicts a third variety of drug carrier, though it is not clear yet whether the MVBs contain either intraluminal vesicles or EVs (segregation hypothesis) or if it is a mixture, or both newly formed intraluminal vesicles and internalized EVs (mixture hypothesis)^[49]^. There are several methods to investigate the endosomal escape of nanoparticles used for drug delivery. In contrast to confocal microscopy, which is not capable of illustrating detailed differentiation, stochastic optical reconstruction microscopy (STORM microscopy) allows the visualization of individual nanoparticles and endosomal escape events using fluorescent colocalization. Wojnilowicz *et al.* were able to quantify individual nanoparticles and categorize between colocalized and non-colocalized glyocoplexes^[50]^. Similarly, Joshi *et al.* managed to visualize the endosomal escape of EVs^[51]^. Moreover, drug carriers in the form of polyplexes could be redirected to the nucleus^[52]^.

### Session I: parasitic EVs

First, **Alberto Ayllon-Hermida** presented his work on the role of EVs in parasite-host interactions between *P. vivax* and human spleen fibroblasts. The group characterized the *P. vivax* spleen-dependent protein 1 by CRISPR-Cas9 knock-in into *P. falciparum* and showed that treatment of spleen fibroblasts with plasma EVs from *P. vivax-*infected patients not only facilitated binding of red blood cells infected with the recombinant protein but also led to upregulation of adhesins.

Then, seven EV-associated protein biomarker candidates for visceral leishmaniasis were proposed by **Ana Torres**. The biomarker candidates were identified from EV preparations from plasma samples of active and cured patients using liquid chromatography-mass spectrometry proteomics, and a validation study is in progress.

Afterward, **Christian Sánchez-López** disclosed the effects of trematode EVs on different liver cell models he observed: The EVs modulated expression of extracellular matrix proteins, regulatory cytokines, and nuclear factor kappa-light-chain-enhancer of activated B-cells (NF-κB) *in vitro*, and of fibrogenic proteins *in vivo* mice livers. Liver fibrosis was not observed in the mouse model, but the findings might shed new light on safety measures when working with parasitic EVs.

The session concluded with **Christophe Wong**, who demonstrated that the choice of methods used for the production and purification of EVs from mesenchymal stem cells can influence their immunomodulatory effect. EVs produced by turbulence showed higher potential for wound healing compared to those produced by starvation, and tangential flow-filtration managed to maintain the potency of the purified EVs, contrary to ultracentrifugation and size exclusion chromatography.

### Session II: infection and interspecies communication

The session addressing infection and interspecies communication started with a presentation by **Ute Westerkamp**. Her focus on EVs in the pathogenesis of Merkel cell carcinoma (MCC) involved a comprehensive multi-OMICS EV characterization. This in-depth analysis of MCC-derived EVs, encompassing their distinct RNA and protein cargos, unveiled potential mechanisms in MCC progression. Notably, the research highlighted the regulatory role of the viral oncoprotein small T in modulating EV protein cargo without affecting the EV messenger RNA (mRNA) cargo.

The succeeding lecture, delivered by **Marie Burt** (née Wiegand), delved into the intricacies of *Klebsiella pneumonia* and its outer membrane vesicles (OMVs). Emphasis was placed on the pivotal role played by OMVs in mediating antibiotic resistance. The research highlighted the capability of OMVs to provide protection against the antibiotic Polymyxin B, extending the protective effects not only to other Klebsiella bacteria but also to diverse bacterial species. However, the study revealed no observed protective effects of OMVs on Gram-positive bacteria.

**Isabel Graf** continued the session by presenting her study on the effects of SARS-CoV-2 infections during pregnancy on placental-derived EVs. Notably, substantial differences were reported based on infection status, with SARS-CoV-2 infection leading to an increase in the quantity of placenta-derived EVs. The alterations observed in the EV RNA cargo suggest that placental EVs could potentially serve as a marker for assessing placental stress after SARS-CoV-2 infections.

**Berit Brusletta** also focused on the RNA profile of EVs in her study, examining plasma EVs isolated from patients during sepsis. Within the context of sepsis, alterations in the characteristics and cargo of plasma EVs become apparent. After thorough characterization, it was observed that, despite patients displaying similar clinical presentations, the isolated EVs showed substantially diverse RNA profiles.

In the following talk, **Rawan Maani** focused on the interaction between colorectal cancer (CRC) EVs and *Escherichia coli* (*E. coli*). Two CRC cell lines were cultured, and EVs were isolated from them. By studying the interplay between CRC-EVs and *E. coli*, they observed that bacteria can lyse EVs and EVs interact with *E. coli* in a disease stage-specific manner. Furthermore, CRC-derived EVs may also influence bacterial phenotypic characteristics such as biofilm formation.

Next, **Giorgia Manni** shared her study about EVs derived from amniotic fluid stem cells. Those EVs are known to have immunoregulatory properties and they were thoroughly characterized by lipidomic, proteomic, and miRNA profiling for this study. *In vivo* experiments with those EVs have revealed that they suppress autoimmune responses by reprogramming dendritic cells.

**Vincenza Tinnirello** shared her insights about the effects of lemon nanovesicles on inflammatory bowel disease. In general, it is shown that plant-derived nanovesicles can exhibit anti-inflammatory effects, which can also be observed *in vivo*. By treating patients suffering from inflammatory bowel disease with lemon nanovesicles, the colon length and architecture can be affected, and pro-inflammatory cytokines are reduced.

In the following talk, **Daniela Yildiz** also focused on inflammatory modulation. The presented study highlights the crucial roles of extracellular proteolysis and EVs in both short- and long-distance communication within the body. They specifically discovered that “a disintegrin and metalloproteinase” (ADAMs) released on exosomes exhibit proteolytic activity, contributing to tissue-destructive effects observed in conditions such as periodontal disease.

To conclude this session, **Fernando del Burgo** highlighted tetraspanins as an immunogenic adjuvant in lipid nanovesicles. A novel vaccine platform was established by integrating liposome technology with the properties of natural EVs, achieved through the addition of tetraspanins to the nanovesicle surface. The study showed that engineered nanovesicles elicited a superior immune response *in vivo* compared to the control without tetraspanins. This advancement holds the potential to streamline future vaccine developments.

### Session III: cardiovascular and metabolic diseases

First, **Christian Sánchez-López** presented his works on the effects of pomegranate juice-derived extracellular vesicles (PgEVs) on acute pancreatitis in mice. Administering PgEVs reduced inflammation, tissue swelling, and pancreatic enzyme activity. It also decreased the activation of a pro-inflammatory pathway and restored antioxidant balance. The findings suggest that PgEVs possess anti-inflammatory and antioxidant properties that could be beneficial in managing acute pancreatitis.

Then, **Nerea Lago Baameiro** explored how EVs from various metabolic tissues exacerbate obesity-related issues and respond to treatments. The group particularly focused on the role of EVs from obese adipose tissue, fatty liver disease, and activated brown adipose tissue (BAT) in promoting thermogenesis. Specifically, they investigated how thermogenic active BAT-EVs induce thermogenesis in non-activated brown adipocytes, offering insights into potential biomarkers and clinical applications for obesity.

Furthermore, **Konxhe Kulaj** disclosed white adipocyte-derived extracellular vesicles (AdEVs) as stimulants for insulin secretion in early-stage prediabetes. AdEVs from obese mice were found to significantly enhance insulin secretion by transferring specific proteins to β-cells, influencing insulinotropic pathways and improving glucose tolerance in mice. This suggests that AdEVs play a role in priming β-cells for increased insulin secretion, potentially aiding in maintaining normal glucose levels during the early stages of prediabetes.

Finally, **Akhil Antony Konkoth** showed the investigation of microvesicles (MVs) derived from various cell types and their impact on coagulation and fibrinolysis, referred to as the “coagulolytic balance”. MVs from stimulated monocytes and endothelial cells displayed high procoagulant activity, resulting in denser and more stable clots, while MVs derived from neutrophils exhibited profibrinolytic properties. In conditions such as sepsis, differences in the activities of these MV subsets may contribute to hemostatic imbalances that affect thrombotic conditions.

### Session IV: fundamental EVs biology

**Alina Milici** started this session by explaining how extracellular lipid vesicles were able to induce Ca^2+^ signals in target cells. After a stimulus, an ionic influx leads to action potential or release of substances by the cell. By studying transient receptor potential (TRP) channels with a focus on TRPA1, the group could show that lipid vesicles were able to trigger changes in intracellular Ca^2+^ concentrations and thus activate TRPA1 channels.

The uptake of EVs by cells is a physiologically relevant process that enables intercellular communication. However, it is complex and depends on different variables like EV composition and type and the receptor cell. Using CRISPR/Cas9, **Miguel Palma-Cobo** identified potential molecular candidates regulating EV uptake. NGS revealed that knocked-out genes for members of the COMMANDER complex were significantly different in comparison to the control. The hits were validated using flow cytometry and bioluminescence assays.

EV surface proteins are crucial mediators of EV cargo delivery. **Maria Laura Tognoli** mapped surface proteins by mass spectrometry and identified proteins by small interfering RNA (siRNA) screening in co-culture using the CROSS-FIRE system. The tetraspanins CD9 and CD63 were identified to be dispensable for EV-mediated cargo transfer. At the time of the talk, blocking assays for validation of the siRNA screening hits for their involvement in EV-mediated uptake and cargo transfer were ongoing.

**Omnia Elsharkasy** presented her work on the identification of genes that play a role in EV-mediated RNA delivery using EVs from MDA-MB-231 cells and HEK293T receptor cells transfected with integrin beta 1 (ITGB1) siRNA. It was shown that ITGB1 and MDA-MB-231-EVs were trafficked together in HEK293T cells, suggesting that ITGB1 plays a role in EV uptake.

To understand the release of small EVs (sEVs), **Krizia Sagini** investigated the molecular machinery by using siRNA screening based on radiolabeled cholesterol for the identification of proteins involved in sEV release. A depletion of synaptosome-associated protein 29 (SNAP29) resulted in a reduction of sEVs carrying classical exosomal markers while not affecting MVB morphology and fusion with the plasma membrane. However, SNAP29 depletion induces a redistribution of MVB towards the cell nucleus. These findings suggest that SNAP29 plays a role in the release of exosomes.

**João Vasco Ferreira** used exosomal lysosome-associated membrane protein 2A (LAMP2A) loading of cargo (e-LLoC) to show that loading of KFERQ-containing proteins on exosomes involved both LAMP2A and heat shock cognate 71 kDA protein HSC70. Furthermore, mass spectrometry of exosomes, early endosomes, and late endosomes revealed that LAMP2A is potentially involved in endosomal organization. The findings suggest that LAMP2A plays a role beyond targeting proteins into exosomes^[53]^.

### Session V: cancer immunity

**Alessandro Sarcinella** presented the impact of tumor-endothelial cell-derived extracellular vesicles (TEVs) on metastatic spread using a murine model of triple-negative breast cancer (TNBC). Injecting TEVs into mice led to immune suppression, observed through increased immune checkpoint expression, and impaired T-cell function in the lungs, spleen, and bone marrow. *In vitro* experiments further demonstrated TEV-induced immune alterations, and *in vivo* findings suggested that TEVs enriched in mTOR support granulocyte-colony stimulating factor (G-CSF) release, contributing to tumor immune suppression and metastasis growth.

**Elisa Costanzo** reported on how colorectal cancer-derived extracellular vesicles (CRC-SEVs) impact liver metastasis by influencing hepatocytes in the formation of a pre-metastatic niche. The study demonstrated that CRC-SEVs prompt hepatocytes to undergo changes associated with fibrosis, inflammation, and immunosuppression, affecting crucial aspects of the pre-metastatic niche. Understanding these effects could contribute to the development of improved diagnostic and therapeutic strategies for the early treatment of liver metastatic disease.

**María Ángeles de Pedro** explored the potential of secretome from menstrual blood-derived stromal cells (S-MenSCs) as a therapeutic approach for tumor-associated macrophages (TAMs). They isolated EVs from this secretome and treated TAMs derived from ovarian cancer patients. The results showed a shift in TAMs towards a more pro-inflammatory M1-like phenotype after treatment with S-MenSCs, suggesting a potential therapeutic avenue for modulating the tumor environment by targeting TAMs.

**Diana Huber** investigated how plasma-derived sEVs from head and neck squamous cell carcinoma (HNSCC) patients affect macrophages and explores the influence of HPV infection in HNSCC. sEVs increased NF-κB activation in macrophages, and the study revealed reversible effects by using NF-κB inhibitors. The research highlights the potential therapeutic targeting of tumor-associated macrophages through sEV modulation in the tumor microenvironment, emphasizing the importance of considering human papillomavirus (HPV) status in patient treatments.

**Carolina Dias** presented her study on pancreatic ductal adenocarcinoma (PDAC) and the exploration of new strategies, specifically sensitizing tumors to immunotherapy due to limited treatment options and poor survival rates. Using genetically engineered mouse models, the study found that impairing PDAC EV secretion unexpectedly accelerated tumor onset, attracting pro-tumorigenic immune responses. Targeting myeloid-related protein 8 in this subset of PDAC tumors might offer a novel therapeutic approach by modulating the immune response and potentially enhancing patient survival.

### Session VI: EVs in cancer progression

**Jamal Ghanam** reported that chromatinized DNA in sEVs from acute myeloid leukemia (AML) blasts, containing histones and S100 proteins, modified the proliferation of bone marrow mesenchymal stem cells (BM-MSCs). The EV-chromatin downregulated p53-mediated transcription of p21 through the increase of mouse double minute 2 homolog (MDM2) expression, which could be reversed by the addition of the MDM2 inhibitor Siremadlin or MDM2 siRNA.

**Venkatesh Kumar Chetty** found Y-box binding protein 1 (YBX1) to be highly upregulated in sEVs from both AML patients and the AML cell line MV4-11, as well as in BM-MSCs treated with these AML cell line derived EVs. Differentiation of BM-MSCs was influenced by MV4-11 EVs but could be rescued when downregulating YBX1 in the MV4-11 cell line, which indicates that YBX1 in AML EVs disrupts normal hematopoiesis in the bone marrow.

A link between breast adipose tissue-derived EVs and the metabolic dysregulation of estrogen receptor (ER) positive breast cancer cells was described by **Alberto Benito-Martin**. The proliferation of ER^+^ cell lines increased after treatment with EVs from breast adipose tissue from overweight or obese women compared to treatment with EVs from lean women, which might be due to the enrichment of several proliferation-promoting miRNAs in obese EVs.

**Matilde Rodriguez Chacón** compared the miRNA cargo of EVs from primary cultures of periprostatic adipose tissue (PPAT) to those from perivesical adipose tissue (PVAT) and found nine EV-miRNAs to be differentially regulated, sharing the retinoic acid receptor Related Orphan Receptor A (RORA) as a common target. The EVs were taken up by prostate cancer cell lines, and functional *in vitro* analysis confirmed the regulation of RORA by the identified EV-miRNAs, resulting in dysregulation of proliferation and inflammation, and linking PPAT with prostate cancer aggressiveness.

**Enrique Baston** investigated the consequence of DNA damage in melanoma cells on the DNA associated with their EVs and its effect on recipient cells. He found that the amount of DNA and the phosphorylated histone y-H2AX was higher in EVs from cells with DNA damage, and that uptake of those EVs resulted in increased DNA damage repair in melanocytes and tumor formation in a mouse model.

A novel approach to treat drug-resistant ovarian cancer was presented by **Juan Antonio Fafián Labora**. He demonstrated that EVs derived from ferroptotic cells with high levels of Fe^2+^ are able to induce ferroptosis in ovarian cancer cells and that these cells, in turn, produce more ferroptotic EVs, resulting in secondary ferroptosis paracrine transmission.

**Malika Singh** highlighted the potential of EVs in anti-cancer drug delivery, showing that incubating human breast cancer cell-line (MDA-MB-231 and T47D) EVs with curcumin resulted in improved curcumin solubility. The therapeutic effects of free curcumin compared to that attached to EVs are currently under investigation.

The role of EV-associated miRNAs in T-cell acute lymphoblastic leukemia (T-ALL) was unraveled by **Tommaso Colangelo**, who found NOTCH1-dependent miRNAs, mostly members of the miR-17-82a cluster and paralogues, enriched in patient EVs. *In vitro*, those miRNAs rescued T-ALL cell proliferation and regulated genes characterizing patients with relapsed early T-cell progenitor ALL, suggesting that EV-miRNAs can alter the heterogeneity and dynamics of the disease in response to therapy.

### Session VII: EVs towards the clinic in cancer

**Vincenzo Verdi** highlighted the significant roles of tumor-derived EVs in various aspects of cancer progression but noted gaps in understanding their *in vivo* functions. The discussion revolved around the development of a novel approach using a Chimeric Antigen Recognition (CAR) strategy to selectively capture and accumulate circulating prostate cancer-derived EVs at chosen cellular targets or tissues. Through redirecting these EVs, the study aimed to gain insights into their roles in processes such as pre-metastatic niche formation, demonstrated in both *in vitro* and *in vivo* experiments using zebrafish embryos.

**Dapi Menglin Chiang** showed that head and neck squamous cell carcinoma (HNSCC) removal surgery effectively eliminated most tumor-related EVs found in both peripheral and local biopsies. By pairwise comparison, PDGFB levels were notably elevated in the two plasma EVs groups before the operation. PDGFB originating from plasma EVs might support tumor growth. Overall, PDGFB within blood EVs could serve as a potent biomarker during HNSCC ablation, aiding in determining the necessary extent of surgical intervention.

**Yoon-Kyoung Cho** presented a study introducing a novel method to digitally detect tumor-derived EVs in the blood plasma samples of lung cancer patients. This method utilizes charge-induced fusion with molecular beacon-encapsulated liposomes within picolitre-sized droplets. The system streamlines detection by eliminating labor-intensive sample preprocessing, confirming fusion through various analyses, and accurately identifying specific mutations such as miR-21 and EGFR L858R/T790M in tumor-derived EVs. Overall, this approach offers a sensitive and simplified means of cancer detection, efficiently identifying tumor-associated mutations while avoiding extensive sample preprocessing.

**Tayfun Tatar** described exosomal miRNA content as a diagnostic tool for glioblastoma using two Gene Expression Omnibus datasets. Analysis revealed distinct miRNA profiles between glioblastoma and controls in both datasets, with machine learning successfully distinguishing between them. The findings propose exosomal miRNA profiling as a non-invasive alternative for early-stage glioma detection, indicating the potential for automated diagnosis using bioinformatics and machine learning techniques.

**Kerstin Menck** disclosed the potential of tumor-associated EVs as predictive markers for immunotherapy in non-small cell lung cancer (NSCLC). By isolating and characterizing sEVs and large EVs (lEVs) from NSCLC patients' blood, they found a notable enrichment of PD-L1 on lEVs compared to sEVs, distinguishing NSCLC patients from controls. Higher levels of PD-L1^+^ lEVs in the blood correlated with improved response to immunotherapy, surpassing the predictive value of tissue PD-L1 expression, particularly in patients with low or absent tissue PD-L1 expression, highlighting PD-L1^+^ lEVs as a promising predictive marker for NSCLC immunotherapy.

**Linda Hofmann** conducted a study focusing on the detection of HNSCC using saliva-derived sEVs and compared their miRNA profiles with plasma-derived sEVs. They identified potential liquid biomarkers, including the top 10 miRNA targets: miR-1245b-5p, miR-1271-5p, miR-432-5p, miR-506-5p, miR-508-3p, miR-517a-3p, miR-519c-3p, miR-548ah-5p, and miR-593-3p. Saliva sEVs from HNSCC patients carried specific immune checkpoints and tumor antigens, showing promise for diagnosis and disease-free survival prediction. The identified tumor-exclusive miRNAs in both saliva and plasma sEVs hold potential as diagnostic and potentially prognostic panels for HNSCC, pending validation in larger cohorts for clinical implementation.

### Session VIII: EVs in liquid biopsy

**Ana Flores-Chova** explained how she used RNA-Seq of exosomal non-coding RNA to identify potential therapeutic targets of renal damage in systemic lupus erythematosus patients. Biological pathway analysis revealed an increasement of miR-16-5p levels in EVs associated with promising targets such as fibroblast growth factors. Furthermore, a significant increasement in exosomal miR-16-5p levels was found.

After renal transplantation, the graft status needs to be monitored by invasive organ biopsy after the transplantation. **Marta Clos-Sansalvador** presented how a non-invasive graft status monitoring through urinary EVs using digital droplet PCR could serve as an alternative. A combined model including glomerular filtration rate, miRNAs, and the expression of vitronectin increased the specificity for detection of interstitial fibrosis and tubular atrophy and suggested that patients can be identified better and stronger without the need for a biopsy. Moreover, urinary EVs are a feasible platform for clinically applicable biomarkers in kidney diseases.

Targeting EV-based diagnostic tools, **Olga Martinez-Arroyo** also investigated a clinical application of plasma EVs for renal damage in systemic lupus erythematosus patients. For this, plasma EV surface antigens were evaluated by flow cytometry. The results suggest that an EV-based signature reflecting the immune inflammatory response could be used as a non-invasive tool for diagnosing renal damage in systemic lupus erythematosus patients.

**Marina Herrero** presented the results of her investigation of the small RNA-secretome in Huntington’s Disease (HD) neurons. While the characterization of neuronal EVs obtained from HD and a control revealed no differences in size and morphology, extracellular small RNAs such as miRNA were significantly downregulated in HD compared to the control. It was suggested that these deregulated exosomal RNAs could have a paracrine and autocrine toxic role in the disease.

Joining virtually, **Laura Varela** presented her work on synovial fluid-derived EVs as a source for potential biomarkers in horse osteoarthritis. An integrated multi-omics approach of proteome and metabolome analysis revealed that the lipid and proteomic profiles of the synovial fluid EVs transform gradually with disease progression and indicated an interrelationship between specific lipids and proteins.

**Estefanía Lozano-Andrés** explained how plasma descending lipoprotein particles can influence quantitative and qualitative EV analysis. She found that the colloidal matrix of the biofluid can influence EV properties such as buoyant density, size and/or refractive index, which could have consequences for downstream EV analysis. Concluding it was highlighted that the interaction of EVs and lipoprotein particles should be considered for EV biomarker profiling.

### Session IX: Therapeutic opportunities of EVs

**Kimberly Schell** discussed Graft versus Host disease, a severe complication following stem cell transplantation that affects a considerable number of patients and frequently results in elevated mortality rates. Due to significant side effects associated with steroid treatment, there is a growing interest in exploring alternatives such as Extracorporeal Photopheresis (ECP). Research on serum EVs in pediatric patients receiving ECP demonstrated distinct variations in EV size and microRNA composition between complete responders and partial/non-responders, suggesting a potential role for miRNA biomarkers in predicting ECP response and providing valuable insights into its mechanisms.

**Krisztina Németh** highlighted the hurdles encountered by therapeutic EVs remaining in circulation, primarily due to swift liver elimination and the presence of a protein corona enveloping them. Research utilizing labeled EVs in mice unveiled their tendency to accumulate in the liver, indicating varying uptake by different liver cells based on EV size. Despite the ApoB corona showing no impact on liver uptake, it significantly diminished EV accumulation in the spleen, showcasing the protein corona's influence on biodistribution. The study indicates diverse uptake of EVs by liver cell types depending on size and sheds light on how protein coronas can modify EV properties.

**Malgorzata Czystowska-Kuzmicz** showed the immunomodulatory effects of placenta MSC-derived EVs compared to parental cells in a human *in vitro* cystic fibrosis 3D-model using a co-culture of primary pseudostratified epithelium cells, small airway fibroblasts, and alveolar macrophages. Demonstrating the potential of this organo-typical co-culture model, historically used in inhalation toxicology, it offers a valuable tool to explore the therapeutic capabilities of MSC-derived EVs under standardized, *in vivo*-like conditions, facilitating precise dosing via aerosol-based delivery for optimized *in vivo* experiments and suggesting its potential for developing similar models to study EV-cell interactions in various human tissues.

**Beatriz Salinas** proposed the utilization of Lutetium-177 (Lu-177) labeled sEVs derived from goat milk for targeted radiotherapy, leveraging their tumor-targeting abilities to deliver radiation specifically to tumors. Two radiolabeling strategies were tested, demonstrating successful labeling with high purity; notably, DOTA-sEVs exhibited stability over time and showed hepatobiliary metabolism observed *in vivo*. The research successfully showcased the development of a novel vectorized nanoradiotherapy using Lu-177 labeled sEVs derived from goat milk, prompting forthcoming experiments to evaluate its biological impact in tumor models.

### Session X: manufacturing EVs

The session about manufacturing EVs started with a presentation from **Elżbieta Karnas**, in which she explored the molecular composition and functional impact of EVs derived from human induced pluripotent stem cells (hiPSC-EVs). The findings indicate that hiPSC-EVs carry bioactive miRNA cargo, differing from their parental cells. These EVs modulate signaling pathways in cord blood-derived hematopoietic stem and progenitor cells at both protein and gene expression levels.

**Yanis Mouloud** focused on clonal mesenchymal stromal cells as it was shown that the deriving EVs have a high therapeutic potential for the treatment of patients with treatment-resistant graft-versus-host disease. As mesenchymal stromal cells tend to easily become senescent, clonal immortalized mesenchymal stromal cells have been developed. EVs from these mesenchymal stromal cells have been attributed with a comparable therapeutic potential with the benefits of increased reproducibility and scalable manufacturing of EV-therapeutics.

Next, **Yvan Courageux** shared his study, which explored Wharton’s Jelly mesenchymal stromal cell-derived EVs produced under Good Manufacturing Practice (GMP) compliance in both 2D and 3D microcarrier-based bioreactors. The isolation of EVs was accomplished through tangential flow filtration and size exclusion chromatography. Despite the 3D culture generating fewer EVs, they exhibited enhanced potency and demonstrated improvements in cardiac function *in vivo*.

**Marieke Roefs** addresses challenges in using mesenchymal stromal cell EVs for therapeutic purposes. By extending the replicative life span and differentiation capacity of mesenchymal stromal cells through human telomerase reverse transcriptase (hTERT) overexpression, reproducible and scalable EV production can be achieved. Telomerized mesenchymal stromal cells from various tissues produced EVs with similar biological activity, miRNA expression patterns, and surface marker expression, suggesting their safe and promising use for standardized EV production in age-associated diseases and tissue regeneration.

In the last talk of this session, **Maria Cardona-Timoner** investigated the promotion of bone regeneration by combining fibrinogen scaffolds with EVs secreted by Dendritic Cells (DCs). Results demonstrated that the incorporation of DC-EVs into fibrinogen scaffolds significantly improved mesenchymal stromal cell recruitment compared to scaffolds alone. Additionally, mesenchymal stromal cells cultured on fibrinogen scaffolds modified with Magnesium exhibited enhanced osteogenic differentiation.

### Session XI: engineering EVs

**Diego Baranda Martínez-Abascal** engaged in the optimization of the EV-protein tagging. For this, he investigated three loading strategies (interior soluble with the affimer fused to the domain of the N-Syntenin, exterior membrane loading with the affimer fused to the CD63 2nd loop and interior membrane loading with the affimer fused to the CD63 C-terminus) and found that all tested constructs led to effective loading of the affimer. However, interior membrane loading resulted in nearly 90% higher affimer loading and an almost 10-fold increase in EV yield.

**Mohamed Elbeltagy** presented how the immunomodulatory potential of immortalized mesenchymal stroma cell-EVs can be enhanced. The immunomodulatory effects of mesenchymal stroma cell-EVs are attributable to a conversion of pro-inflammatory extracellular adenosine phosphate into anti-inflammatory adenosine. In the study, the group introduced the coding region of CD39 into clonally expanded immortalized mesenchymal stroma cells using lentiviral vectors. They could show that the engineered EVs were co-expressing CD39 and CD73 and that their immunomodulatory potential *in vitro* was amplified by the CD39 overexpression.

In contrast, **Tobias Tertel** used a lentiviral bases CRISPR/Cas9 technology to knock out the coding regions of CD73 and CD81 to gain a better understanding of the underlying mechanisms for the immunomodulatory potential of mesenchymal stroma cells. The characterization of EV subpopulations revealed no phenotypic changes. Furthermore, CD81-KO-EVs showed no activity in a multi-donor mixed-lymphocyte reaction assay, while CD73-KO-EVs surprisingly retained their immunomodulatory ability. Overall, the results indicate that CRISPR-Cas9 gene knockout is an effective method for studying the roles of EV-associated molecules.

**María Iranzo Martínez** developed therapeutic EVs that can be used to transport Cas9 ribonucleoproteins to target cells to restore the function of the type 7 collagen protein by removing the mutated collagen type VII alpha 1 chain COL7A1 exogen 80. This system was developed for recessive dystrophic epidermolysis bullosa patients. However, due to its versatility, it can be adapted to other pathologies where gene therapy using Cas9 ribonucleoproteins to knock out/inactivate a specific gene is a therapeutic option.

**Paula Heredia** described how genetically modified anti-CD19-chimeric antigen receptor (CAR)-T cells released exosomes with cytotoxic activity against CD19+ cancer cells. Furthermore, the group found by bicinchoninic acid assay that T-cells modified to overexpress hepatocyte growth factor-regulated tyrosine kinase substrate were producing more exosomes in Jurkat cells.

### Session XII: advances in EV methodology

**Andreu Matamoros-Angles** presented a mechanical, non-enzymatic approach for the isolation of EVs from brain tissue, which kept EV surface markers intact and might be beneficial for researchers of neurodegenerative diseases. Proteomic analysis showed no difference in intraluminal proteins compared to brain-derived EVs isolated with collagenase, but revealed differential expression of proteins potentially in the EV corona and membrane.

A novel affinity-bead-based EV isolation method was demonstrated by **Roberto Frigerio**, utilizing Membrane Sensing Peptides that interact hydrophobically with small diameter particles enclosed by a membrane with high curvature. This method allowed for a traceless release of EVs and achieved higher yield and purity than several conventional methods for EV isolation from serum, plasma, and urine.

**Teresa Valero** disclosed another affinity-based approach, which is highly customizable. The presented method allowed high flexibility regarding the choice of nanoparticle, tag, affinity ligand, linker, and downstream analysis method.

**Maria-Anthi Kakavoulia** described an EV-labeling method for flow cytometry using galectins that bind glycan structures on EV surfaces. The galectin-based staining approach was demonstrated for mesenchymal stem cell-derived EVs and compared to staining with tetraspanin antibodies (CD9, CD63, CD81), showing that CD63 was not present on al galectin-positive EVs.

A novel detection method for EVs in their native biological samples using digital holographic microscopy combined with particle tracking was presented by **Fredrik Eklund**. EVs are labeled without prior purification with gold nanoparticles, which can be used in excess, since single gold nanoparticles and unlabeled molecules do not interfere with the phase contrast detection method.

**Marije Kuipers** proposed using cell coverage area as a normalization factor to compare EV-targeting to cells. The strategy was demonstrated with different cell types (osteoblasts, osteoclasts, monocytes, and prostate cancer cells), and cell coverage area was calculated using light microscopic imaging to determine each cell type-specific EV binding and uptake efficiency.

### MOVE fellows

The symposium was closed with a mixed session given by eight students receiving funding from the MOVE Fellowship Program. **Valentina Moccia** presented her work performed in Utrecht (Netherlands) about the establishment of an EV-isolation protocol for *Manila clam* hemolymph, a bivalve often used for aquaculture and as an environmental bioindicator.

During her stay in Antwerp (Belgium), **Natalia Valle-Tamayo** collaborated with Yael Hirschberg and used LC-MS/MS-based proteome profiling to identify potential biomarkers beyond amyloid-β and τ. In combination with further EV characterization using NTA and cryo-electron microscopy, different clinical stages of Alzheimer’s might be explored.

Additionally, **Yael Hirschberg** investigates EVs to differentiate between various dementia diseases, such as Alzheimer's, Parkinson’s, and Lewy body dementia. EVs from the cerebrospinal fluid are used and characterized with typical dementia markers.

**Martin Wolf** collaborated with the group of Samir el Andaloussi at Karolinska Institute in Stockholm (Sweden) to explore the protein corona using flow cytometry. The study uncovered the significance of the protein corona in influencing EV functions, yet highlighted that ultracentrifugation has the capability to disrupt it.

**Sergio Garcia Garcia** went to Torino (Italy), where he studied MSC-EVs in chronic kidney disease (CKD). Currently, there is no effective treatment for CKD, but EVs have led to promising results. His findings support the potential clinical translation of EV-based therapies for CKD.

**Genevieve Anghileri** also stayed in Utrecht (Netherlands) to investigate matrix-bound vesicles and secreted EVs in early osteogenesis. The primary objective was to elucidate the composition and role of these vesicles in mineral formation, aiming to discern their precise mode of action.

During her stay in Madrid (Spain) commencing in January 2024, **Mireia Gomez-Duro** will be investigating the novel EV subtype CD133 in triple-negative breast cancer. CD133 is a pentaspan membrane glycoprotein that can serve as a stem cell and cancer stem cell marker. It is not suitable as a prognostic marker and is rather associated with chemotherapy resistance in different epithelial cancer types.

**Monica Guarro** went to Eindhoven (Netherlands) to characterize mRNA loading onto mammal EVs using STORM microscopy. Utilizing various loading strategies (incubation, freeze-thawing, sonication, electroporation, and extrusion), she found that freeze-thawing appears to be the best method for mRNA loading while electroporation and extrusion lead to a degradation of the EVs.

## CONCLUSION

Overall, the 1st MOVE symposium in Málaga provided a highly prosperous event for interactions among the international and young EV scientists from 16 European EV societies. With the warm and sunny weather in Málaga last October, the city in the south of Spain turned out to be the right choice for our event. The selection of the scientific session topics covered almost all fields of the current and potential future EV research areas, from methodological improvements, over fundamental biological EV topics, up to general physiological, disease, or cancer-related aspects. Several sessions were introduced by renowned keynote speakers and concluded with short talks from young enthusiastic EV scientists. All sessions were followed by intensive and fruitful discussions, which were carried out after each talk, during the meeting breaks and even beyond during the social events in the evening. Moreover, each session consisted of not only oral presentations but also poster presentations. Their large number would go beyond the scope of this summary report, so it is unfortunately not possible to discuss them in detail, but they are, of course, also deserving of particular thanks. With its high number of driven and well-educated EV scientists, the young European research community appears to be perfectly equipped for future challenges in EV research.

Some highlighted talks, reviews, and poster presentations are summarized and presented in the EVCNA special issue “EV Insight” which you are currently reading.

The international MOVE organization board, particularly **María Yáñez-Mó**, **Bernd Giebel**, **Charlotte Lawson** and **Benedetta Bussolati**, on behalf of the 16 participating European EV societies, thanks all speakers, all poster presenters, all scientific contributors, the generous international sponsors, and the local Spanish organizing team, especially **María Isabel Lucena**, **Juan Manuel Falcón-Pérez**, **Eduardo García Fuentes** and **Aurélie Papineau**, for their efforts in making the 1^st^ MOVE Symposium such a success. The basic MOVE idea, to establish future European collaborative research projects by supporting young scientist mobility, worked very well. The profit of this first MOVE event will be re-invested in the MOVE Fellowship Program, our PhD student travel scholarship fund, to foster multiple new bilateral EV research cooperations all over Europe.

We are all looking forward to our following MOVE meetings, which we will organize together. A second MOVE symposium is already scheduled from 8th - 11th October 2024 and will be hosted by Maja Kosanović, Department for Immunology and Immunoparasitology of the University of Belgrade, Serbia. The authors hope that we will meet again this autumn in Belgrade!

## DECLARATIONS

### Copyright

© The Author(s) 2024.
